# Patients’ Stem Cells Differentiation in a 3D Environment as a Promising Experimental Tool for the Study of Amyotrophic Lateral Sclerosis

**DOI:** 10.3390/ijms23105344

**Published:** 2022-05-11

**Authors:** Eveljn Scarian, Matteo Bordoni, Valentina Fantini, Emanuela Jacchetti, Manuela Teresa Raimondi, Luca Diamanti, Stephana Carelli, Cristina Cereda, Orietta Pansarasa

**Affiliations:** 1 Department of Brain and Behavioral Sciences, University of Pavia, Via Forlanini 6, 27100 Pavia, Italy; eveljn.scarian@mondino.it (E.S.); v.fantini@golgicenci.it (V.F.); 2 Cellular Models and Neuroepigenetics Unit, IRCCS Mondino Foundation, via Mondino 2, 27100 Pavia, Italy; matteo.bordoni@mondino.it; 3 Laboratory of Neurobiology and Neurogenetic, Golgi-Cenci Foundation, Corso San Martino 10, 20081 Abbiategrasso, Italy; 4 Department of Chemistry, Materials and Chemical Engineering “Giulio Natta”, Politecnico di Milano, Piazza Leonardo da Vinci 32, 20133 Milan, Italy; emanuela.jacchetti@polimi.it (E.J.); manuela.raimondi@polimi.it (M.T.R.); 5 Neuroncology Unit, IRCCS Mondino Foundation, via Mondino 2, 27100 Pavia, Italy; luca.diamanti@mondino.it; 6 Pediatric Clinical Research Center “Fondazione Romeo ed Enrica Invernizzi”, Department of Biomedical and Clinical Sciences “L. Sacco”, University of Milan, Via Giovanni Battista Grassi, 74, 20157 Milan, Italy; stephana.carelli@unimi.it; 7 Genomic and Post-Genomic Unit, IRCCS Mondino Foundation, via Mondino 2, 27100 Pavia, Italy; cristina.cereda@asst-fbf-sacco.it; 8 Neonatal Screening and Metabolic Disorders Unit, Department of Woman, Mother and Neonate, “V. Buzzi” Children’s Hospital—ASST Fatebenefratelli Sacco, Via Lodovico Castelvetro, 52, 20154 Milan, Italy

**Keywords:** ALS, 3D bioprinting, neural stem cells, motor neurons progenitors, motor neurons

## Abstract

Amyotrophic lateral sclerosis (ALS) is a neurodegenerative disease (NDD) that affects motor neurons, causing weakness, muscle atrophy and spasticity. Unfortunately, there are only symptomatic treatments available. Two important innovations in recent years are three-dimensional (3D) bioprinting and induced pluripotent stem cells (iPSCs). The aim of this work was to demonstrate the robustness of 3D cultures for the differentiation of stem cells for the study of ALS. We reprogrammed healthy and sALS peripheral blood mononuclear cells (PBMCs) in iPSCs and differentiated them in neural stem cells (NSCs) in 2D. NSCs were printed in 3D hydrogel-based constructs and subsequently differentiated first in motor neuron progenitors and finally in motor neurons. Every step of differentiation was tested for cell viability and characterized by confocal microscopy and RT-qPCR. Finally, we tested the electrophysiological characteristics of included NSC34. We found that NSCs maintained good viability during the 3D differentiation. Our results suggest that the hydrogel does not interfere with the correct differentiation process or with the electrophysiological features of the included cells. Such evidence confirmed that 3D bioprinting can be considered a good model for the study of ALS pathogenesis.

## 1. Introduction

Neurodegenerative diseases (NDDs) are a group of debilitating pathologies that affect over 50 million people around the world, with an increasing rate due to the progressive ageing of the population. Despite their different features, which cause disparate manifestations, they all lead to the death of specific neural cells. Unfortunately, the causes of these pathologies are still unknown, and they remain incurable [1,2,3].

Amyotrophic lateral sclerosis (ALS) is an NDD that affects upper and lower motor neurons (MNs), causing weakness, muscle atrophy and spasticity. It can be manifest in two forms, the sporadic form (sALS), which constitutes 90–95% of cases, and the familiar form (fALS), which constitutes 5–10% of cases, and can involve different causative genes. As for other types of NDDs, such as Alzheimer’s and Parkinson’s disease, there are only symptomatic treatments available [4,5,6]. 

Due to the lack of cure for these diseases, it is even more important to find valid experimental models for the study of their etiopathogenesis. In recent years, many experimental models, such as fruit fly, mouse, nematode worm and baker’s yeast [7,8,9,10,11,12], but also traditional 2D cell cultures [7,13], have been used. In this field, one of the most recent and important newnesses is the use of induced pluripotent stem cells (iPSCs). These are pluripotent stem cells obtained from adult somatic cells using specific transcription factors called “Yamanaka factors”. iPSCs can be differentiated in many types of cells, such as neurons, astrocytes and oligodendrocytes. As they can be obtained from patients, they allow scientists to create a more realistic pathological model and to advance the discovery of novel specific treatments in the field of personalized medicine [14,15,16].

Another important innovation in recent years is three-dimensional (3D) bioprinting. It consists of the inclusion of different types of cells in various biocompatible biomaterials to create a bioink, which is then printed with the use of a bioplotter. 3D bioprinting allows the growth and viability of the included cells in a 3D environment, which can better mimic the biological characteristics of the tissues or organs. Moreover, it allows the study of the interaction between cells, and between cells and the environment.

Since its definition by Guillemot in 2010 [17], many biomaterials have been developed and used for the generation of 3D constructs, such as hydrogels, i.e., hyaluronic acid, xyloglucan, collagen, alginate sodium, gelatin, nanofibers and carbon-based biomaterials [18,19,20,21].

To date, a large amount of progress has been made in the field of 3D bioprinting, specifically, the creation of vessels, bones, cartilages and heart cells [22,23,24,25,26]. The nervous tissue is relatively new to the topic of 3D bioprinting, and to date, progress has also been made in the creation of small structures, such as nerve grafts and retinal ganglion, but progress in disease modelling is limited [27,28,29].

The aim of this work was to join the field of iPSC differentiation and 3D bioprinting, demonstrating the validity of 3D cultures for the differentiation of cultured stem cells in order to study NDDs in a more realistic model. After a first mechanical characterization of the hydrogel, we developed a protocol of both control (CTRL) and sALS stem cells differentiation in 3D structures, and we compared the differentiation processes of these cells in 2D and 3D through immunofluorescence analysis, confocal microscopy and Real-Time qPCR (RT-qPCR). Moreover, we used a specific antibody for the detection of cells’ action potentials to test the electrophysiological characteristics of printed NSC34. We concluded that the inclusion and printing of cells allow good viability and differentiation of healthy and sALS stem cells, confirming 3D bioprinting as an optimal tool for the study of ALS pathogenesis.

## 2. Results

### 2.1. Hydrogel Characterization Confirmed Its Optimal Features for Cells Cultures

To evaluate the mechanical features of the printed structures we performed three different assays, measuring the moisture, swelling ratio and porosity of both printed constructs composed only of Cellink Bioink (IK-102000; Cellink, Gothenburg, Sweden) and constructs composed of the hydrogel and culture medium.

The water content, evaluated using the moisture test, is an important characteristic of the integrity of hydrogels. We found that in both types of constructs, within or without a culture medium, the moisture percentage was above 95% without significant differences (Figure S1). Swelling is directly linked to moisture, which indicates the ability to absorb water. In both types of constructs, after three hours of submersion, the swelling ratio reached a plateau at approximately 55% for the constructs composed of the hydrogel and the medium, and approximately 40% for the constructs composed only of the hydrogel (Figure S2). Moreover, in both cases, and at each time point, the mass swelling ratio did not exceed the value of 2 (Table S1). Finally, we tested the porosity, which is related to the amount of pore space of the hydrogel. The porosity test showed that the printed constructs composed of Cellink Bioink and culture medium had a higher porosity percentage when compared to constructs composed only of hydrogel (Figure S3).

### 2.2. Neural Stem Cells (NSCs) Showed Good Viability during the 3D Differentiation Process

**Figure 1:**
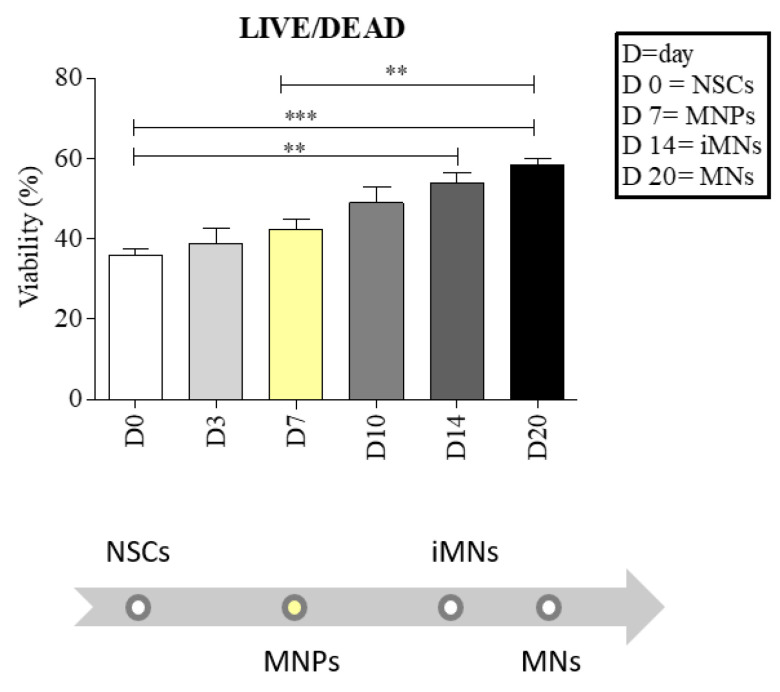
Neural stem cells (NSCs) maintain good viability during the differentiation to motor neurons (MNs). The cell viability was evaluated at days 0, 3, 7, 10, 14 and 20 of differentiation. At day 7, the cells reached the stage of motor neuron progenitors (MNPs), and the viability was increased by 18%. The MNPs bar is in yellow because it is the first stage of cell specialization to MNs. At day 14, MNPs reached the stage of immature motor neurons (iMNs) with an increase in viability of 50%, whereas at day 20, MNPs reached the stage of MNs with an increase in viability of 62%. There is a statistically significant difference between NSCs and iMNs (** *p* < 0.01), between NSCs and MNs (*** *p* < 0.001) and between MNPs and MNs (** *p* < 0.01). Error bars indicate SD. Data were analyzed using ANOVA, followed by the Bonferroni test (GraphPad Prism 8).

To evaluate the capability of the cells to proliferate in 3D conditions, we used the LIVE/DEAD viability assay, and multiple measures were taken from three different 3D structures, printed at 25 °C with a pressure between 45 and 70 kPA, in each differentiation step [18]. sALS NSCs encapsulated and printed into the bioink showed good viability during the differentiation process. We observed an increasing viability when NSCs were printed and differentiated from the bioink, with statistically significant differences between NSCs and immature motor neurons (iMNs) (day 0 vs. 14, ** *p* < 0.01), between NSCs and motor neurons (MNs) (day 0 vs. 20, *** *p* < 0.001) and between motor neuron progenitors (MNPs) and MNs (day 7 vs. 20, ** *p* < 0.01) (Figure 1).

### 2.3. NSCs Showed Good Differentiation When Printed in 3D

To confirm that the bioink allows the correct differentiation process of NSCs, we performed an immunofluorescence analysis for the typical markers of the differentiation stages.

**Figure 2:**
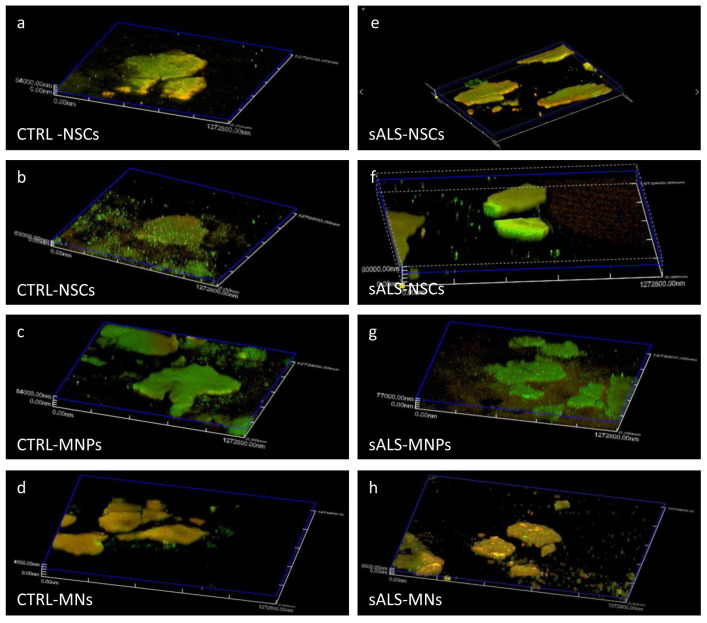
Cells express the typical markers of each stage of differentiation in 3D bioprinting. NSCs differentiated from induced pluripotent stem cells (iPSCs) of control (CTRL), and sporadic amyotrophic lateral sclerosis (sALS) subjects were grown into Cellink Bioink (IK-102000; Cellink, Gothenburg, Sweden) and differentiated into MNPs and MNs. Cells express the typical markers of every differentiation step: (**a**,**e**) NSCs: Nestin = green and SOX2 = red; (**b**,**f**) NSCs: SOX1 = green and PAX6 = red; (**c**,**g**) MNPs: Olig2 = green and PAX6 = red and (**d**,**h**) MNs: TUBB3 = green and Chat = red. The images were taken from videos acquired through confocal microscopy (Olympus Fluoview, FV10i, Tokyo, Japan).

Figure 2 shows an example of the confocal acquisition 3D reconstruction of CTRL and sALS constructs during the cell differentiation process. Cells, at all steps of differentiation, formed colonies and expressed typical markers: Nestin, SOX2, SOX1 and PAX6 for NSCS; Olig2 and PAX6 for MNPs; and TUBB3 and Chat for MNs. The inclusion in the hydrogel and the following printing process did not affect the correct differentiation of cells.

Furthermore, the corrected total cell fluorescence (CTCF) analysis allowed a quantification of the markers’ immunofluorescence in the different layers of the confocal reconstruction. The CTCF identified the layer at approximately 75 μm from the beginning of the structure as the one with the maximum immunofluorescence intensity for almost every differentiation step (Figure S4). Using confocal software (Olympus Fluoview, FV10i, Japan), we obtained the reconstruction video from which the images were acquired, which shows the cell colony and its marker expression in every confocal plane. Moreover, the video shows the thickness of the colony itself (Supplementary Videos), which is impossible to appreciate from 2D cultures images (Figures S5 and S6).

### 2.4. Printed Cells Expressed the Typical Markers of the Differentiation Stages 

**Figure 3:**
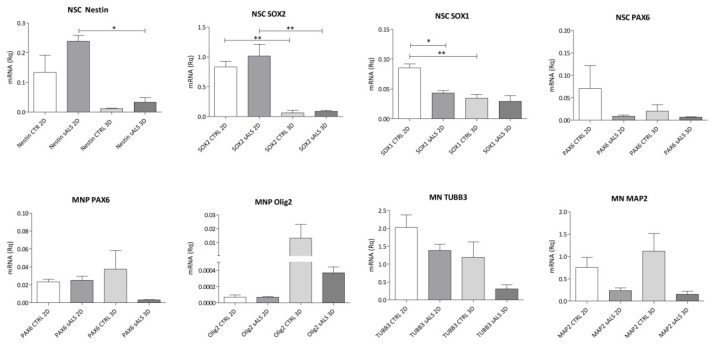
RT-qPCR confirms the expression of the typical markers during differentiation. The differentiation was confirmed using the markers for NSCs (Nestin, *SOX2*, *SOX1* and *PAX6*), MNPs (*PAX6* and *Olig2*) and MNs (*TUBB3* and *MAP2*). Error bars indicate SD. Data were analyzed using ANOVA, followed by the Bonferroni test. * *p* < 0.05; ** *p* < 0.01 (GraphPad Prism 8).

To confirm the correct differentiation and obtain a more precise quantitative measure of markers’ expression, we tested the expression of Nestin, *SOX2*, *SOX1*, *PAX6*, *Olig2*, *TUBB3* and *MAP2* using RT-qPCR. The cells, both from CTRL and sALS, expressed the expected specific set of markers for each differentiation step in both 2D and 3D conditions (Figure 3).

NSCs showed a reduction in Nestin, *SOX2* and *SOX1* expression in 3D structures (* *p* < 0.05 and ** *p* < 0.01) compared to the 2D condition, which is probably due to the printing process. Moreover, both in 2D and 3D, sALS NSCs expressed more Nestin and *SOX2* than CTRL cells, whereas this trend was inverted for *SOX1* and *PAX6*. In the stage of MNPs, we observed a difference between CTRL and sALS cells: in sALS, there was a lower expression of *PAX6* and *Olig2*, especially in 3D, when compared to CTRL cells. Moreover, in both sALS and CTRL, the printing process increased the expression of the MNP marker *Olig2*.

Finally, the number of sALS cells expressing MNs markers, in both 2D and 3D conditions, showed a drastic reduction when compared to CTRL ones. Furthermore, the expression of *MAP2* in CTRL MNs increased, and the number of cells expressing *TUBB3* was reduced in 3D cultures when compared to 2D.

### 2.5. Printed Cells Maintain Their Electrophysiological Characteristics

**Figure 4:**
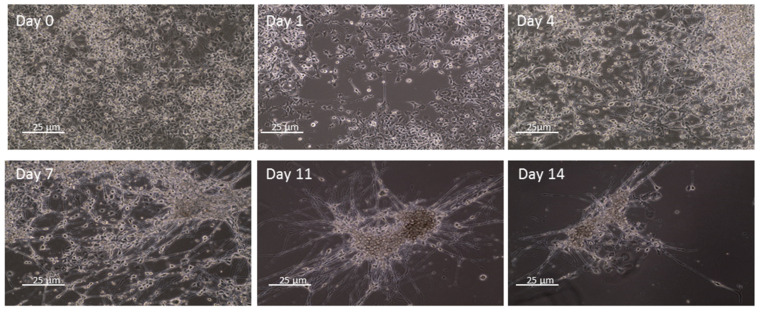
NSC34 differentiate in 14 days of culture. NSC34 were differentiated using DMEM High Glucose, 1% penicillin/streptomycin, 1% L-glutamine and 10 μM all-trans retinoic acid (RA), and the differentiation process was evaluated at days 0, 1, 4, 7, 11 and 14 using the optical microscope Axio Imager 2 (Zeiss, Oberkochen, Germany). Cells began to show propagation already at day 1 and then grouped into colonies with many interconnections. Scale bar = 25 µm.

To test the electrophysiological characteristics of cells included in the hydrogel, we used NSC34 cells, which share many characteristics with human MNs. First, we developed a protocol of NSC34 differentiation at a more mature stage. As shown in Figure 4, already at day 1, after the beginning of differentiation, cells began to display propagations. At day 14, cells changed their morphology and appeared aggregated in colonies with the growth of many propagations and connections between themselves.

**Figure 5:**
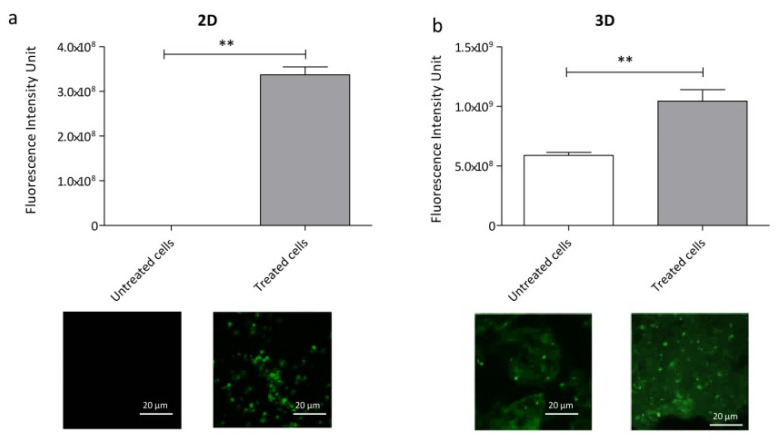
NSC34 cells maintain their electrophysiological characteristics when printed. NSC34 cells were induced to fire with 15 mM KCl both in 2D (**a**) and 3D (**b**). Then, an immunofluorescence analysis using the neuronal marker c-fos was performed. Data show that the hydrogel does not interfere with the firing capacity of printed NSC34. Error bars indicate SD. Data were analyzed using a *t*-test followed by the Mann–Whitney test. ** *p* < 0.01 (GraphPad Prism 8). Scale bar = 20 µm.

After 14 days of differentiation, we performed an immunofluorescence analysis using the neuronal activity marker c-fos. Cells were first treated with 15 mM KCl to induce the firing. As shown in Figure 5, both the 2D- and 3D-cultured cells increased their firing after treatment.

The fluorescence intensity was higher in 15 mM KCl-treated cells when compared to the non-treated cells (** *p* < 0.01). Moreover, the analysis of immunofluorescence intensity demonstrated that the inclusion in the gel and the printing process did not interfere with the electrophysiological characteristics of NSC34.

We noticed that, while in the 2D cultures, non-treated NSC34 did not fire, untreated 3D NSC34 showed a low firing intensity, probably due to the autofluorescence of the bioink, which caused background fluorescence.

## 3. Discussion

NDDs are a huge problem for society, not only due to the difficulties that they cause for patients, but also due to their economic and social costs. For these reasons, the study of these diseases has rapidly developed in recent years, and many in vitro and in vivo models have been studied [7,8,9,10,11,12,13,30].

In recent years, two main fields have emerged in the study of models for NDDs: the use of iPSCs and their differentiation for the development of a more patient-like model, and 3D bioprinting. Three-dimensional bioprinting is an emerging technique that allows the creation of 3D culture models, in which cells can grow in an environment similar to the physiological one. Many studies have focused on the use of 3D bioprinting, but few have dealt with NDDs [15,16,18,19].

In this work, we demonstrated the possibility to grow and differentiate iPSCs-derived NSCs in a 3D environment, in order to create a more physiological model for the study of NDDs, such as ALS.

First of all, we performed mechanical tests on our printed constructs. Moisture is an important value for hydrogels, inasmuch as it indicates the water content, but it also correlates with the capacity to maintain dimension stability. Moreover, moisture facilitates the solubility and diffusion of substances [31,32]. We found a moisture percentage above 95% in both the printed construct types (the one composed only of hydrogel and the one composed of both cell medium and hydrogel in a 1:10 proportion), as previously reported [32,33], indicating a high amount of water content in both the conditions. A precedent study demonstrated that the water content can depend on the presence of hydrophilic groups in the hydrogel itself [33], which, in our hydrogel, are represented by the hydroxyl groups located on the surface of the cellulose fibres [34] and by the intrinsic hydrophilic nature of sodium alginate [35]. The swelling characteristics of the hydrogel are directly linked to moisture. Swelling is the ability of the hydrogel to absorb water. A high swelling rate indicates that the hydrogel can easily change shape and break in a hydrated environment. Moreover, crosslinking can affect and decrease the swelling ratio, reducing the empty spaces available to water molecules within the hydrogel [36]. We found that the printed structures composed of only the hydrogel had a lower swelling rate when compared to the one composed of hydrogel and culture medium. In both conditions, the swelling ratio reached a plateau at around 3 h after submersion, indicating that it could no longer change shape. Finally, we performed a porosity assay. Porosity plays a huge role in nutrient and oxygen diffusion, especially in an environment without a vascular system [32]. We found that the porosity percentage is higher in structures composed of hydrogel and cell medium.

As few studies have focused on the development of a 3D nervous model, we first performed a LIVE/DEAD assay on sALS NSCs to ascertain the cell viability during the differentiation process. We found that the inclusion in the hydrogel and the printing process did not interfere negatively with the viability of the cells. On the contrary, we observed increased viability after the printing process, with a significant increase between the first stage of differentiation, that of NSCs, and the last stage, that of MNs.

This evidence demonstrated the possibility of using Cellink Bioink (IK-102000; Cellink, Gothenburg, Sweden) for the cultivation of stem cells and their differentiation. We then demonstrated, through confocal microscopy, that cells do not only live and proliferate in the hydrogel but that they correctly differentiate. We found that, in cells from both CTRL and sALS patients cultivated in 3D, the expression of the typical markers of the differentiation steps strongly confirms the correct differentiation process. As opposed to 2D-cultured cells, 3D cultures allow the study of cell organization and how cells connect with one another in a 3D environment, which better mimics the tissue organization. These results further confirm precedent findings, which show that neurons grown in 3D form a functional network that is not achieved in 2D [37,38].

To obtain more precise quantitative data about the differentiation markers, we performed an RT-qPCR. First, we observed that the gel did not interfere with the expression of the typical cell markers, except for NSCs, as already reported for the *ASGR1* gene [39]. The differences between CTRL and sALS cells were maintained when cells were printed in 3D. Moreover, both in 2D and 3D conditions, there was a greater expression of Nestin and *SOX2* in sALS NSCs than in CTRL NSCs, whereas the trend was inverted for *SOX1* and *PAX6*. Nestin is a marker of undifferentiated nervous cells at a stage that precedes the exit of the cell cycle and the cell committee [38], whereas *SOX2* is implicated in self-renewal in undifferentiated cells [40,41]. On the other hand, *SOX1* and *PAX6* are considered markers of a more “mature” stage of stem cells; *SOX1* is reported to represent activated neural stem progenitors and to be the earliest marker of the neuroectodermal lineage [39], whereas *PAX6* is also an MNP marker. Since Nestin and *SOX2* are considered markers of a more “immature” stage of NSCs, our data suggest that sALS NSCs seem to be less differentiated than CTRL cells.

In both 2D and particularly in 3D, we found differences in the expression of specific markers also in MNPs and MNs. We observed a decreased expression of *PAX6* in sALS MNPs compared to CTRL cells. A possible explanation for this is that sALS cells differentiate before CTRL cells, as *PAX6* is also expressed in NSCs, and it could be considered a marker of more immature MNPs. On the contrary, there was a major expression of *PAX6* in CTRL, indicating that there were still immature MNPs present and a mixed population in CTRL with the concurrent presence of immature and mature MNPs. Moreover, we noticed that in both NSCs and MNPs, the expression of *PAX6* was low. It was already demonstrated that embryonic stem cells undergo low to high levels of *PAX6* expression during their differentiation to neural stem cells, fading away when they reach a more differentiated cell type [42]. We observed that in 3D, both in CTRL and sALS, there was an increase in *Olig2* expression, indicating that 3D promotes MNPs maturation. Finally, at the stage of MNs, both in 2D and 3D, we noticed a reduction in ALS cells expressing MNs markers when compared to CTRL ones, as expected from the motor neuronal death characteristic of the ALS pathology. Moreover, we noticed that in CTRL, the 3D condition caused an increase in *MAP2* expression and a decrease in *TUBB3* expression. This indicates a major presence of mature MNs (mMNs) in 3D cultures when compared to 2D cultures. These findings suggest that the 3D environment increases the differentiation of CTRL MNPs in mMNs when compared to the 2D environment. In fact, precedent studies have suggested that, whereas *TUBB3* is a marker of iMNs [43,43], *MAP2* is a marker of mMNs [44,45].

Ultimately, we evaluated the possibility of cells maintaining their electrophysiological characteristics when printed. To investigate this aspect, we used NSC34 cells, a mouse spinal cord x neuroblastoma hybrid cell line. These types of cells constitute a good model for the study of the nervous system, as they summarize many aspects of MN development in an immortalized system. Moreover, they produce action potentials when stimulated with KCl [46]. We trialled different methods to obtain electrophysiological recordings of the included NSC34, such as the patch-clamp technique and multielectrode array (MEA), without results. These assays were difficult due to the bioink itself, which forms a film around the cells, preventing the adhesion between the cell membrane and the patch pipette or the MEA chip containing the electrodes. For this reason, we performed an immunofluorescence analysis with the use of the antibody c-fos, an action potential marker. We found that the hydrogel did not interfere with the electrophysiological characteristics of stimulated cells. Although the hydrogel caused background fluorescence due to an intrinsic autofluorescence, yet described in other types of hydrogels [47,48], the fluorescence intensity was significantly increased after the treatment when compared to untreated cells, which showed a minor fluorescence intensity, as in 2D conditions. 

In conclusion, the inclusion and the printing in Cellink Bioink (IK-102000; Cellink, Gothenburg, Sweden) allowed not only good viability in the stem cells but also their differentiation in MNs. Moreover, it did not interfere with the electrophysiological characteristics of printed NSC34. This will allow future studies on NDDs and, in particular, on ALS in a more physiological environment, which better mimics the tissue characteristics of patients, such as the spatial organization.

## 4. Materials and Methods

### 4.1. Enrolment of ALS Patients and Healthy Volunteers

Peripheral blood mononuclear cells (PBMCs) were isolated from an sALS patient, diagnosed at the IRCCS Mondino Foundation (Pavia, Italy) and tested for genetic mutations with Next-Generation Sequencing, as described in Filosto et al., 2018 [49]. Patients positive for genetic mutations were excluded from this work. 

One healthy CTRL subject was recruited at the IRCCS Policlinico S. Matteo Foundation in Pavia (Italy). The subject was interviewed on their personal health history to confirm the normal phenotype and avoid the presence of any pathology. 

All individuals signed the consensus after reading informative notes.

### 4.2. Isolation of PBMCs

Total blood was obtained from the sALS patient and the CTRL subject and conserved into EDTA blood tubes. PBMCs were obtained using Ficoll-Histopaque^®^-1077 (Sigma-Aldrich, Milan, Italy) within 24 h, following the manufacturer’s instructions. PBMCs were then collected, and the cell viability was determined via the Trypan Blue (Sigma-Aldrich, Milan, Italy) Exclusion Test using an automatic cells counter (TC20TM Automated Cell Counter, BioRad, Segrate, Italy). PBMCs were then preserved in Fetal Bovine Serum (FBS) (EuroClone, Pero, Italy) and 10% dimethyl sulfoxide (DMSO) (Sigma-Aldrich, Milan, Italy) to prevent cell death and stored in liquid nitrogen. 

### 4.3. PBMCs Reprogramming

PBMCs were reprogrammed following a protocol already set up by our group [18]. In brief, 5 × 10^5^ cells were first suspended in PBMC medium (StemPro™-34 + SCF 100 ng/mL, FLT-3 100 ng/mL, IL-3 20 ng/mL, IL-6 20 ng/mL) and 4 days later transduced with a virus obtained from the CytoTune^®^-iPS 2.0 Sendai Reprogramming Kit (Invitrogen, Carlsbad, CA, USA) using the following formula:

$$Volume\;\;\left(\mu L\right)=\frac{MOI\;\left(CIU/cell\right)\cdot n.\;\;of\;cells}{titer\;of\;virus\;\left(CIU/\mathrm{mL}\right)\cdot \;10^{-3}\left(\mathrm{mL}/\mu L\right)}$$

where *MOI* is a virus-dependent number; KOS = 5; hc-Myc = 5; hKlf4 = 3; and the titer of virus is a lot-dependent number.

After 24 h of incubation, the medium was replaced with the PBMC medium. Cells were then plated on a vitronectin-coated culture dish and cultured for 7 days in complete StemPro™-34 medium (ThermoFisher Scientific Inc., Waltham, MA, USA) and then in Essential 8™ Medium (ThermoFisher Scientific Inc., Waltham, MA, USA) until the formation of iPSCs colonies. The colonies were manually picked and grown on vitronectin for 3–5 passages. iPSCs were conserved in Essential 8™ Medium and 10% DMSO in liquid nitrogen. 

### 4.4. iPSC Differentiation in NSCs 

The obtained iPSCs were then differentiated from NSCs in 2D. iPSCs growth on vitronectin at a confluence of 60–70% was cultured in an NSC Differentiation Medium composed of Neurobasal (ThermoFisher Scientific Inc., Waltham, MA, USA) and Neural Induction Supplement 50X (ThermoFisher Scientific Inc., Waltham, MA, USA) for 7 days. The medium was changed every other day and at day 8 exchanged with an Expansion Medium composed of Neurobasal 2X (ThermoFisher Scientific Inc., Waltham, MA, USA), Advanced DMEM/F12 2X (ThermoFisher Scientific Inc., Waltham, MA, USA) and Neural Induction Supplement 50X (ThermoFisher Scientific Inc., Waltham, MA, USA).

### 4.5. Characterization of the Hydrogel

Cells were encapsulated in Cellink Bioink (IK-102000; Cellink, Gothenburg, Sweden), printed using the Bioplotter Cellink BioX (Cellink, Gothenburg, Sweden) and crosslinked using the provided crosslinking agent (Cellink, Gothenburg, Sweden). Cellink Bioink is a commercial bioink composed of cellulose nanofibrils and alginate. Bioink was printed using a 0.41 mm nozzle (23G) at 25 °C, with a printing speed of 600 mm/min and with a pressure between 45 and 70 kPa.

#### 4.5.1. Moisture

The moisture was evaluated, as described by Shawan et al. [32], and modified using the method described by Piola et al. [33], for both constructs composed of only the hydrogel and of cell medium DMEM Low Glucose (Carlo Erba, Cornaredo, Italy) and hydrogel in a 1:10 proportion. After 3 days in distilled water, hydrated constructs were weighed and dried for 3 days at 37 °C. The percentage of moisture was evaluated using the following formula:

$$Moisture\left(\%\right)=\left[\frac{W^{H}-W^{D}}{W^{H}}\right]\;\times \;100$$

where *W^H^* is the weight of hydrated prints before drying and *W^D^* is the weight of the constructs after drying. The experiment was replicated three times with three different samples for each condition.

#### 4.5.2. Swelling Test

The swelling test was performed, as described by Asohan et al. [36] and Piola et al. [33]. 

Printed constructs, both constructs composed only of the hydrogel and of cell medium and hydrogel in a 1:10 proportion, were dried for 2 days at 37 °C and then weighed. They were rehydrated in distilled water and weighed again after the water was removed at different time points (0, 1, 3, 6 and 24 h). The swelling ratio (*S*) was calculated using the following formula:

$$S=\left[\frac{W^{H}-W^{D}}{W^{D}}\right]\times 100$$

where *W^H^* is the weight of hydrated prints and *W^D^* is the weight of dehydrated constructs. The experiment was replicated three times with three different samples for each condition.

#### 4.5.3. Porosity

The porosity of Cellink Bioink was evaluated by using the liquid displacement method, as described by Piola et al. [33]. We evaluated the porosity of both bioprinted constructs composed of only hydrogel and constructs composed of cell medium and the hydrogel in a 1:10 ratio. A known quantity of absolute ethanol (Carlo Erba, Cornaredo, Italy) was weighed, and the constructs were submerged. After 5 min of submersion, samples were weighed and at the end the ethanol was weighted after the removal of the printed constructs.

The porosity was calculated using the following formula:

$$Porosity\left(\%\right)=\left[\frac{W_{1}-W_{3}}{W_{2}-W_{3}}\right]\times 100$$

where *W*_1_ is the weight of pure ethanol, *W*_2_ is the weight of ethanol in combination with the printed construct and *W*_3_ is the ending weight of ethanol without the construct. 

The experiment was replicated three times with three different samples for each condition.

### 4.6. Printing of NSC Cell Line

NSCs differentiated from iPSCs were encapsulated in sterilized Cellink Bioink (IK-102000; Cellink, Gothenburg, Sweden) and printed using the Bioplotter Cellink BioX (Cellink, Gothenburg, Sweden). This bioink has already been used for the encapsulation and growth of human NSCs, as reported by Chiang et al. [50]. For the encapsulation process, 6 × 10^5^ cells/mL NSCs were mixed with the bioink in a 1:10 proportion using two 5 mL syringes and a Luer connector. The solution composed of the bioink and cells was then printed using the parameters already set in our laboratory [18] and following the manufacturer’s recommendation (BPR-IK-102000, Cellink, Gothenburg, Sweden). Briefly, encapsulated cells were printed using a 0.41 mm nozzle (23G) at 25 °C, with a printing speed of 600 mm/min and a pressure between 45 and 70 kPa. We used an open-source Computer-Aided Drafting (CAD) software to design an STL grid model (FreeCAD©, ver. 0.16), and the slicing process was assessed using Slic3r (version 1.2.9). The printed 3D constructs were then crosslinked using the ionic crosslinking agent provided with the bioink (Cellink, Gothenburg, Sweden). The printed NSCs were cultivated in the Expansion Medium described above. 

### 4.7. Differentiation of NSCs

**Figure 6:**
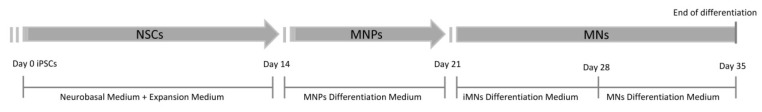
Workflow for MN differentiation. Induced pluripotent stem cells (iPSCs) were cultured for 14 days for the differentiation in NSCs and their expansion. At day 14, the medium was exchanged with MNP Differentiation Medium, which allowed the formation of MNPs in 7 days. MNs then formed in 14 days with the use of two different media, a medium for the formation of iMNs (iMN Differentiation Medium) and another one for their maturation (MN Differentiation Medium).

NSCs in 2D and the corresponding cells included and printed in the bioink were cultivated in appropriate media for the differentiation first in MNPs and then in MNs (Figure 6).

NSCs were plated on a vitronectin-coated plate for the 2D condition, or printed in the bioink for the 3D condition, and cultivated for 7 days with the MNP Differentiation Medium, composed of Neurobasal 2X, Advanced DMEM/F12 2X, Neural induction supplement 50X, 0.1 µM Retinoic Acid (Sigma-Aldrich, Milan, Italy) and 0.5 µM Purmorphamine (ThermoFisher Scientific Inc., USA). 

MNPs were cultivated for 7 days in a medium composed of Neurobasal 2X, Advanced DMEM/F12 2X, 0.5 µM Retinoic Acid, 0.1 µM Purmorphamine, 10 ng/mL GDNF (ThermoFisher Scientific Inc., USA), 10ng/mL IGF (ThermoFisher Scientific Inc., USA) and 10 ng/mL BDNF (ThermoFisher Scientific Inc., Waltham, MA, USA) to obtain iMNs. iMNs were then cultivated for 7 days in the same medium of MNPs with the addition of 0.1 µM Compound E (Santa Cruz Biotechnology, Inc., Santa Cruz, CA, USA) for the complete differentiation in mMNs, as previously described [51]. 

### 4.8. Viability Assay

Due to the impossibility of performing the Trypan Blue exclusion method on the printed cells, due to the hardening of the constructs by the crosslinking agent, the viability was evaluated using the LIVE/DEAD Viability Assay (ThermoFisher Scientific Inc., USA), which has already been used in different 3D bioprinting studies [52,53,54,55]. Constructs were incubated in 0.75 μM calcein AM (Invitrogen, Carlsbad, CA, USA) (green) and in 1 μM ethidium homodimer-1 (Invitrogen, Carlsbad, CA, USA) (red), dissolved in cold 1X PBS (Sigma-Aldrich, Milan, Italy). Calcein stained living cells, indicating intracellular esterase activity, while ethidium homodimer-1 indicated a loss of plasma membrane activity, staining dead cells. After 45 minutes of incubation, they were washed with 1X PBS and observed using a microscope Axio Imager 2 (Zeiss, Oberkochen, Germany), with an Axiocam Mrm camera (Zeiss, Oberkochen, Germany).

Viability was evaluated at days 0, 3, 7, 10, 14 and 20.

### 4.9. Immunofluorescence Analysis

Cells cultured in 2D and 3D constructs were first fixed in 4% paraformaldehyde (ThermoFisher Scientific Inc., Waltham, MA, USA), for 15 (2D) and 30 min (3D) and blocked in 5% Normal Goat Serum (ThermoFisher Scientific Inc., Waltham, MA, USA) and 0.1 % tween, for 1 h at room temperature (RT) and for 2 h at 4 °C, respectively. They were both incubated in primary antibody at 4 °C overnight (Table 1). For NSC immunofluorescence, we combined four markers: Nestin, a type VI intermediate filament protein expressed in the cytoskeleton and in the cells during development [40,56]; SOX2 and SOX1, transcription factors involved in self-renewal maintenance [41,57] and neurogenesis [58,59], respectively; and finally, PAX6, a transcription factor present during embryonic development [60]. The same analysis was performed for MNPs and differentiated MNs with the typical markers of the specific stages: Olig2, involved in MN and oligodendrocyte differentiation [61] and PAX6 for MNPs; TUBB3, involved in neurogenesis and axon guidance and maintenance [62]; and Chat, which catalyzes the biosynthesis of the neurotransmitter acetylcholine [63], for MNs.

The following day, they were incubated with the secondary antibody (Table 1): 1 h at RT for the 2D cultures and 3 h at 4 °C for the 3D constructs. Finally, the 2D cultures were mounted with Prolong^®^ Gold anti-fade reagent DAPI (Invitrogen, Carlsbad, CA, USA) and the 3D structures were mounted with Polyvinyl alcohol mounting medium with DABCO^®^ anti-fading (Sigma-Aldrich, Milan, Italy), then dried and nail-polished. Samples were acquired using confocal microscopy (Olympus Fluoview, FV10i, Tokyo, Japan), and an image was acquired every 15 μm. Three-dimensional reconstructions were performed using the relative software (Olympus Fluoview, FV10i, Tokyo, Japan). Image analysis was carried out using the Fiji-ImageJ free software (https://imagej.net/Fiji/Downloads, accessed on 13 January 2021).

Moreover, immunofluorescence quantification was performed for every confocal slice of the 3D structure. The corrected total cell fluorescence analysis (CTFC) was calculated on the whole cell colony and obtained according to previously described protocols using the following formula [64,65]:

CTCF = Integrated Density − (Area of selected cell × Mean fluorescence of background readings)

The CTFC of the first layer was normalized as 1.0, and the relative fluorescence intensity of each other layer was defined as the ratio of the layer CTFC to that of the first layer, as described by Xu and colleagues in 2019 [66].

### 4.10. RT-qPCR

Total RNA from NSCs, MNPs and MNs, cultured both in 2D and 3D conditions, was extracted using TRIzol^®^ (Invitrogen, Carlsbad, CA, USA) following the manufacturer’s instructions. RNA quality and quantity were evaluated using the NanoDrop spectrophotometer (Invitrogen, Carlsbad, CA, USA), and 1000 ng of RNA was reversed-transcribed using an iScriptcDNA Synthesis Kit (BioRad, Segrate, Italy) following the manufacturer’s instructions. Finally, a quantitative polymerase chain reaction (qPCR) was performed using the CFX Connect™ Real-Time PCR Detection System (BioRad, Segrate, Italy) and the SYBR Green Master Mix (BioRad, Segrate, Italy). Then, 1 μL of cDNA was used, and the expression of all the genes (Table 2) was run at 60 °C.

### 4.11. Electrophysiological Analysis

Cells were first differentiated in 2D using a medium composed of DMEM High Glucose (Carlo Erba, Cornaredo, Italy), 1% penicillin/streptomycin (Carlo Erba, Italy), 1% L-glutamine (Carlo Erba, Cornaredo, Italy) and 10 μM all-trans retinoic acid (RA) (Sigma Aldrich, Italy) for 14 days, and the differentiation was evaluated using the optical microscope Axio Imager 2 (Zeiss, Oberkochen, Germany) at days 0, 1, 4, 7, 11 and 14. For the 3D sample, 1 × 10^6^ NSC34 cells were then encapsulated in the bioink. Cells were treated with 15 mM KCl, as NSC34 cells do not fire action potentials autonomously, and the treatment with KCl induced them to fire. Experiments were driven simultaneously in 2D and 3D and both with cells treated with KCl for 20 min at 37 °C and with cells left in 1X PBS for 20 min at 37 °C as a control sample. After the treatment, an immunofluorescence analysis with the antibody c-fos (Santa Cruz Biotechnology, Santa Cruz, CA, USA; dilution 1:300) was performed. C-fos is an indirect marker of neuronal activity often expressed when neurons fire action potentials. C-fos is transcripted transcribed when the neuronal activity is strong enough to activate the mitogen-activated protein kinase (MAPK) signalling pathway, which has a low calcium sensitivity. External stimuli cause a rapid transcription of this gene [67]. Cells were then analyzed using confocal microscopy (Olympus Fluoview, FV10i, Tokyo, Japan).

### 4.12. Statistical Analysis

All tests were performed three times, and data were presented as the mean ± SD. Statistical analyses were performed using GraphPad Prism (GraphPad Prism 8), adopting the *t*-test followed by Mann–Whitney test for comparison between two groups, and one-way analysis of variance test (ANOVA), followed by the Bonferroni test, in the experiments with more than two groups. A *p*-value < 0.05 was considered statistically significant.

## Figures and Tables

**Table 1:** Primary and secondary antibodies used for the immunofluorescence analysis.

Primary Antibody	Secondary Antibody	Brand
Nestin 1:100	CFTM 594 goat anti-mouse 1:500	Abcam (Cambridge, UK)
SOX2 1:250	CFTM 488A goat anti-rabbit 1:500	Proteintech (Chicago, IL, USA)
SOX1 1:200	CFTM 488A goat anti-rabbit 1:400	Abcam (Cambridge, UK)
PAX6 1:100	CFTM 594 goat anti-mouse 1:400	Invitrogen (Carlsbad, CA, USA)
Olig2 1:400	CFTM 488A goat anti-rabbit 1:500	Novus Biologicals (Centennial, CO, USA)
TUBB3 1:400	CFTM 488A goat anti-rabbit 1:500	Abcam (Cambridge, UK)
Chat 1:200	CFTM 594 goat anti-mouse 1:400	Novus Biologicals (Centennial, CO, USA)

**Table 2:** List of RT-qPCR primers used for the detection of the differentiation step markers.

Gene	Primer Sequences (5′-3′)	Annealing Temperature
**Nestin**	**F** GGA AGA GAA CCT GGG AAA GG **R** GAT TCA GCT CTG CCT CAT CC	60 °C
***SOX2***	**F** AGTCTCCAAGCGACGAAAAA **R** TTTCACGTTTGCAACTGTCC	60 °C
***SOX1***	**F** AAATACTGGAGACGAACGCC **R** AACCCAAGTCTGGTGTCAGC	60 °C
***PAX6***	**F** TGTGTGCTCTGAAGGTCAGG **R** CTGGAGCTCTGTTTGGAAGG	60 °C
***TUBB3***	**F** CAGATGTTCGATGCCAAGAA **R** GGGATCCACTCCACGAAGTA	60 °C
***MAP2***	**F** AGGGCTGGTAGGTTGGATCT **R** TGTGTCTCTGCCTTTGTATC	60 °C
***GAPDH***	**F** CAG CAA GAG CAC AAG AGG AAG **R** CAA CTG TGA GGA GGG GAG ATT	60 °C

## Data Availability

The datasets generated and/or analyzed during the current study are available upon reasonable request to the corresponding author in the Zenodo repository, doi:10.5281/zenodo.5838093, accessed on 30 January 2022.

## Supplementary Materials

The following supporting information can be downloaded at: https://www.mdpi.com/article/10.3390/ijms23105344/s1.
