# Supplementary material for: Patients’ Stem Cells Differentiation in a 3D Environment as a Promising Experimental Tool for the Study of Amyotrophic Lateral Sclerosis
Source: Int J Mol Sci. 2022 May 11;23(10):5344. doi: 10.3390/ijms23105344 (PMC9141644; doi:10.3390/ijms23105344)
Supplement: Supplementary file 1 [file ijms-23-05344-s001.zip › Supplementary Figures.pdf]

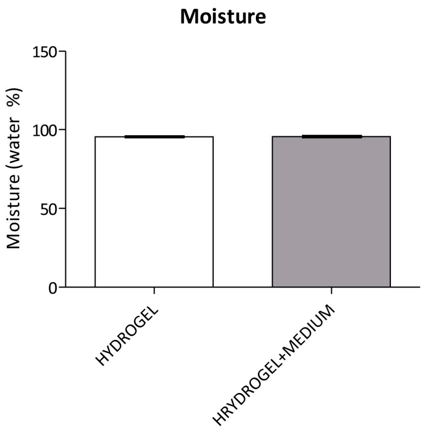

Figure S1: Moisture percentage in printed constructs. Moisture percentage was evaluated in both constructs composed of only Cellink Bioink (Cellink Life Sciences, Sweden) and in constructs composed of the hydrogel and culture medium. In both the cases moisture percentage was above 95% (95.46% for the first type of constructs and 95.55% for second one) without significant differences. The experiment was replicated three times with three different samples for each condition. Data were analyzed by ANOVA, followed by Bonferroni test. Error bars indicate SD (GraphPad Prism 8).

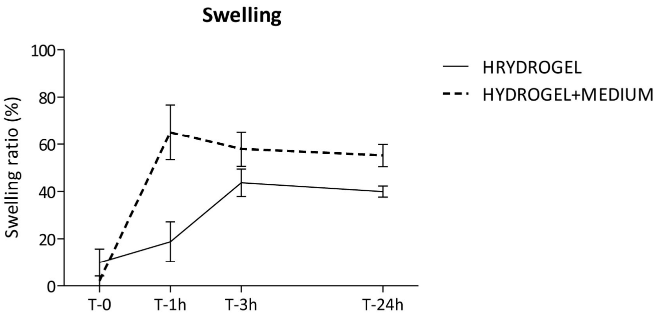

Figure S2: Swelling ratio in printed constructs. Swelling ratio was evaluated in both constructs composed of only Cellink Bioink (Cellink Life Sciences, Sweden) and in constructs composed of the hydrogel and culture medium. In both the cases the swelling ratio reaches a plateau after three hours (T-3h) in distilled water. The experiment was replicated three times with three different samples for each condition. Data were analyzed by ANOVA, followed by Bonferroni test. Error bars indicate SD (GraphPad Prism 8).

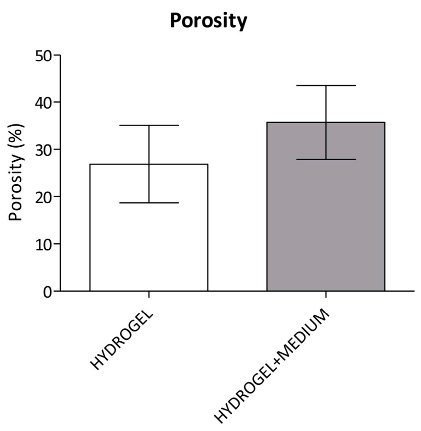

Figure S3: Porosity percentage in printed constructs. Porosity percentage was evaluated in both constructs composed of only Cellink Bioink (Cellink Life Sciences, Sweden) and in constructs composed of the hydrogel and culture medium. In constructs composed only by hydrogel the porosity reaches a value near 27%, whereas the porosity increases to 36% in the constructs composed of hydrogel and cells medium. The experiment was replicated three times with three different samples for each condition. Data were analyzed by ANOVA, followed by Bonferroni test. Error bars indicate SD (GraphPad Prism 8).

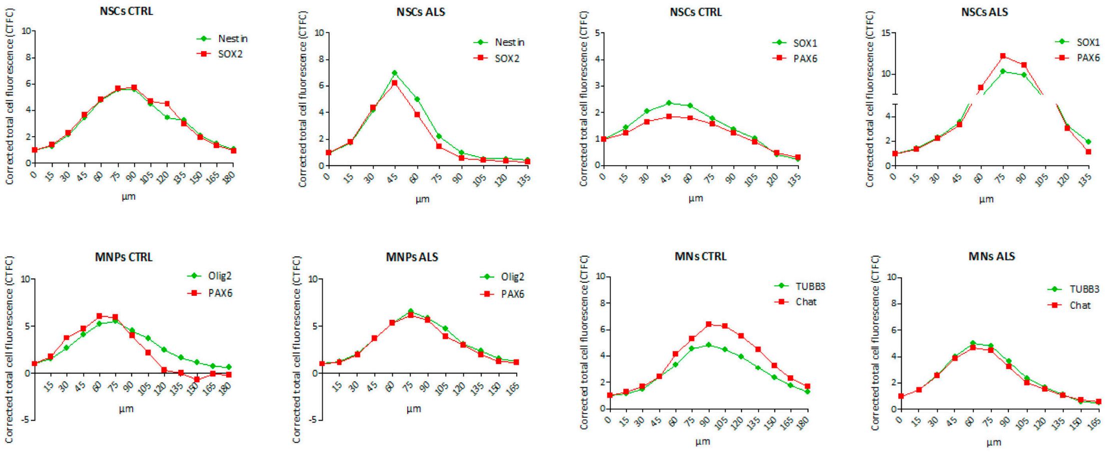

Figure S4: Corrected total cell fluorescence (CTFC) in 3D constructs layers. To quantify NSCs, MNPs and MNs markers for every layer of the constructs, CTFC was calculated. The CTFC of the first layer was normalized to 1. In every condition, NSCs, MNPs and MNs, and in both CTRL and ALS, the CTFC reach a maximum value around 75µm from the beginning of the structure.

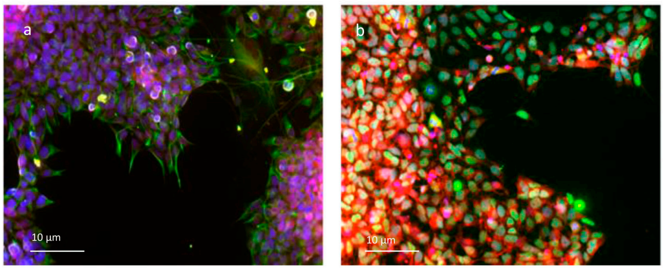

Figure S5: Immunofluorescence of 2D NSCs. NSCs express the typical markers of this step of differentiation: a) Nestin= green and SOX2=red; b) SOX1= green and PAX6= red. Nuclei were stained with DAPI (blue). Scale bar=10µm.

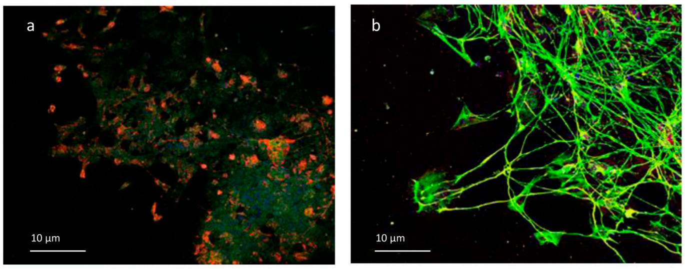

Figure S6: Immunofluorescence of 2D MNPs and MNs. MNPs and MNs express the typical markers of the differentiation steps: a) MNPs: Olig2= green and PAX6= red; b) MNs :TUBB3=green and Chat= red. Nuclei were stained with DAPI (blue). Scale bar=10μm.
