# Supplementary material for: Patients’ Stem Cells Differentiation in a 3D Environment as a Promising Experimental Tool for the Study of Amyotrophic Lateral Sclerosis
Source: Int J Mol Sci. 2022 May 11;23(10):5344. doi: 10.3390/ijms23105344 (PMC9141644; doi:10.3390/ijms23105344)
Supplement: Supplementary file 1 [file ijms-23-05344-s001.zip › Supplementary Table S1.pdf]

Table S1: Mass swelling ratio of printed structures

Type of construct	Mass swelling ratio
Cellink Bioink T-0h<br>Cellink Bioink + Culture Medium T-0h	1.10<br>1.02
Cellink Bioink T-1h<br>Cellink Bioink + Culture Medium T-1h	1.19<br>1.65
Cellink Bioink T-3h<br>Cellink Bioink + Culture Medium T-3h	1.44<br>1.58
Cellink Bioink T-24h<br>Cellink Bioink + Culture Medium T-24h	1.40<br>1.55
